# Examining Community Clinicians Use of Imaginal Exposure with Childhood Anxiety Disorders

**DOI:** 10.21203/rs.3.rs-2763135/v1

**Published:** 2023-04-05

**Authors:** Stephen P.H. Whiteside

**Affiliations:** Mayo Clinic

**Keywords:** child, anxiety disorders, treatment, community, imaginal exposure, dissemination

## Abstract

Although community therapists rarely use exposure when treating youth with anxiety disorders, they are more likely to endorse the use of imaginal exposure, relative to in vivo or interoceptive exposure. Such use of imaginal exposure could reflect a sub-optimal replacement for in vivo exposure or a targeted intervention to address anxiety-provoking thoughts, such as in generalized anxiety disorder. The current study used the survey responses of 178 community-based clinicians to examine these competing hypotheses. The results indicated that therapist endorsement of more clearly defined imaginal exposure was significantly lower than other techniques such as cognitive restructuring or that imaginal exposure was other used instead of more intensive forms of exposures. In addition, most of the endorsed interventions were targeted at helping youth cope with anxiety rather than corrective learning. Alternatively, there was no observed association between imaginal exposure and treatment of general anxiety. These finding suggest that community therapist use of imaginal exposure is suboptimal and unlikely to improve treatment outcomes.

## Introduction

Anxiety disorders are one of the most common mental health diagnoses in childhood, tend to persist over time, and lead to impairment and additional psychopathology (Bittner et al., 2007; Ezpeleta et al., 2001; Kessler et al., 2005). Cognitive behavioral therapy (CBT) is the most well-supported treatment for childhood anxiety disorders (CADs; Wang et al., 2017), with growing evidence and expert consensus that exposure is the most essential active ingredient of treatment (Stewart et al., 2016; Whiteside, Sim, et al., 2020). Unfortunately, most community-based clinicians do not provide exposure when working with CADS (Whiteside et al., 2022; Whiteside, Deacon, et al., 2016; Whiteside, Sattler, et al., 2016) even in response to direct training. However, community clinicians tend to be more likely to use imaginal exposure (rather than in vivo or interoceptive exposure) and to increase use with training (Whiteside et al., 2022; Whiteside, Deacon, et al., 2016). Since this use of imaginal exposure has not led to better outcomes (Whiteside et al., 2022), it is unclear whether use of imaginal exposure is an insufficient application of exposures or if failure to improve outcomes reflects the inadequacy of the treatment protocol because therapists were appropriately using imaginal exposures when indicated to address generalized anxiety disorder (GAD).[1] The current study uses existing survey data to explore whether community clinicians willingness to use imaginal exposure represents sub-optimal use of “exposure-light” or direct exposure targeting anxiety-provoking thoughts, such as in the treatment of GAD. To our knowledge, data to explore this question has not been reported.

Imaginal exposure involves the repeated recitation of anxiety-provoking thoughts, images, or narratives. Through a process of habituation or inhibitory learning, the association between the thoughts and anxiety is thought to diminish resulting in decreased anxiety symptoms (Benito & Walther, 2015). When anxiety-provoking thoughts are the primary symptom of an anxiety disorder, imaginal exposures are the most direct and powerful treatment component. As such, under the labels prolonged exposure or trauma narrative, imaginal exposure is the corner stone of treatment for post-traumatic stress disorder (PTSD; Foa et al., 2005). Similarly, imaginal exposures are the primary modality for treating intrusive thoughts in obsessive-compulsive disorder (OCD), such as unwanted violent or sexual thoughts (Abramowitz, 1996). Although less thoroughly documented in the literature, imaginal exposure, such as to worries of worst-case scenarios occurring, are recommended in the treatment of GAD (Kendall et al., 2005; Whiteside, Brennan, et al., 2020). Imaginal exposures can also be used as a supplement to in vivo exposures to heighten focus on and engagement with in vivo exposures, such as repeating “These germs will make me sick” while touching door handles. Finally, although not typically recommended by experts, imaginal exposures can be used to prepare patients for in vivo exposures, such as imaging giving a speech before progressing to in vivo exposures involving talking in front of an audience.

There are other, non-exposure, therapeutic techniques for responding to anxiety-provoking thoughts based on different models of therapeutic change. For instance, cognitive restructuring involves identifying and challenging thoughts and beliefs that cause anxiety through overestimating the likelihood of negative events (Berman et al., 2015). Cognitive restructuring is often including in CBT as well as cognitive therapy and is based on a model of change in which relief from anxiety comes through correcting cognitive distortions. In contrast, third-wave approaches are based on a model that therapeutic improvement stems from reducing the avoidance and resistance to unpleasant emotional experiences (Twohig et al., 2015). As such these treatment approaches employ acceptance or mindfulness techniques in response to anxiety provoking thoughts. Finally, while not part of research-generated protocols, therapists often report using techniques such as thought-stopping or distraction techniques when treating CADs (Whiteside, Deacon, et al., 2016). Although these techniques are likely not associated with an articulated theory for change, functionally the goal appears to be to reduce anxiety in the moment through avoidance (through suppression or distraction) of anxiety provoking thoughts.

To understand community clinician use of imaginal exposure, it is necessary to examine the context and manner in which they deliver this technique. To begin with, simple endorsement of a short imaginal exposure item (as has been used previously Whiteside, Deacon, et al., 2016) is likely not sufficient to understand if clinicians are employing high quality exposure directed at anxiety provoking thoughts or engaging in suboptimal exposure by using imaginal techniques in lieu of, or perhaps in preparation for, in vivo exposures. Moreover, it is important to understand clinician’s relative use of other techniques. If clinicians spend 10% of session time on imaginal exposure, but 40% of session time (including during exposures) implementing other techniques based on a competing or contradictory mechanism of change, the efficacy of exposure is likely diminished. As such, a better understanding of current community practice is important for appropriately designing dissemination and training efforts that address actual practices that are leading to sub-optimal outcomes.

The current study uses community clinicians’ survey responses to examine whether use of imaginal exposure is better understood as sub-optimal delivery of exposure versus direct targeting of anxiety provoking thoughts in GAD. Specifically, we propose that the sub-optimal exposure theory would be supported if therapists endorsed imaginal exposure less frequently than use of strategies associated with other models of therapeutic change, and if therapists endorsed using interoceptive exposure in lieu in vivo exposure more often than other more stringent uses of exposure. Alternatively, the direct use of imaginal exposure to address GAD worries would be supported if therapist endorsement of imaginal exposure was more strongly associated with the frequency of treating GAD than with other CADs. We hypothesized that therapist’s descriptions would be more consistent with a suboptimal exposure approach, rather than directly targeted to GAD-associated anxiety provoking thoughts.

[1] The observation that community therapist use of imaginal exposure by cannot be assumed to be substandard practice as it may re ect appropriate use with GAD has been raised by multiple reviewers and noted as limitation of previous studies (Whiteside et al., 2022; Whiteside, et. al, under submission).

## Methods

### Participants

The sample is a subset from a previously reported study (Whiteside, Deacon, et al., 2016), limited to the 178 clinicians who reported using in-session imaginal exposure therapy on an outpatient basis for CADs. One hundred and forty-one of the respondents provided their profession, which included doctoral level psychologists (PhD; *n* = 39, 21.9%; PsyD; *n* = 21, 11.8%), social workers (*n* = 53, 29.8%), masters degree licensed counselors (*n* = 17, 9.6%), and marriage and family therapists (*n* = 11, 6.2%). All of the respondents endorsed treating childhood anxiety disorders often and just under half of the sample (*n* = 80; 44.9%) endorsed specializing in childhood anxiety disorders (inclusive of former anxiety disorders OCD and PTSD). Respondents reported practicing for an average for 15.06 years (SD = 10.1, range 1 to 40 years) with 95% practicing for at least three years. Among the 139 clinicians that provided their professional orientation, most generally endorsed two distinct orientations (mea*n* = 1.99, SD = 1.11), most commonly including CBT (88.5%).

### Procedures

Clinicians were identified and recruited through a series of three steps. First, a survey was used to identify therapists who work with anxious children. The survey was emailed directly to all psychologists and social workers who had supplied an email address to the state of Minnesota licensing boards and to all mental health professionals practicing within a regional health system (*N* = 7,273). A link to the survey was also included in email newsletters sent to the members of the statewide professional organizations for psychologists, social workers, and marriage and family therapists (e.g., Minnesota Psychological Association). An initial invitation and two reminders were sent through each channel. The survey inquired about the populations of clients to which the clinician provided treatment. Because the link was included in newsletters it is not known how many clinicians viewed the invitation. A total of 2,869 clinicians responded to the survey.

Second, a follow-up survey about assessment and treatment practices was emailed to the 1,002 clinicians who reported treating anxious youth on the first survey. Responses to the survey were described as confidential because any identifiable information provided by the clinicians was removed by the institution’s survey research center that administered the survey. To keep the survey to a manageable length and based on previous work finding consistent technique endorsement across diagnoses (Hipol & Deacon, 2013), anxiety disorders were grouped together and included separation anxiety, generalized anxiety, social anxiety, specific phobia, panic, and agoraphobia, as well as disorders previously classified under anxiety that are treated with exposure, obsessive compulsive disorder and post-traumatic stress disorder. A total of 331 respondents completed the treatment description measures and were included in the current study. An additional 75 began the survey, but did not complete enough items to be included. A response rate of 33% (331 of 1,002) is comparable to previous surveys (e.g., 27 to 30%; Addis & Krasnow, 2000; Becker et al., 2004).

Third, clinicians from the 331 who completed the follow-up survey were selected based on their responses to two items: a) if they responded “yes” to a general question about whether they provide exposure therapy for child anxiety disorders (*n* = 201) and b) indicated that they use “Exposure to feared mental events” with the therapist present with at least moderate frequency. This resulted in the final sample of 178 clinicians that report using in-session imaginal exposure to treat childhood anxiety disorders.

### Measures

#### Treatment Techniques.

Respondents were first asked to endorse the frequency with which they used a variety of treatment techniques when working with anxious children (0-never used, 1-rarely used, 2-moderately used, 3-often used, 4-always used). The list of techniques and endorsement has been reported previously (Whiteside, Deacon, et al., 2016). For the current study, only seven items describing techniques specifically to address anxious thoughts were selected and are displayed in the appendix.

#### Technique Use During Exposure.

Five items were written for the current study to examine therapist implementation of techniques during exposure (see Appendix). The primary item of interest was the frequency with which therapists reported encouraging patients to repeat anxiety provoking thoughts during exposure. Four additional items corresponded to the other thought-based treatment techniques (separating from symptoms, cognitive restructuring, distraction, and thought stopping) during exposure. Items were rated on a 5-point Likert-type scale ranging from 0 (never use) to 4 (always use).

#### Exposure Therapy Delivery Scale (ETDS; Deacon et al., 2023).

The ETDS is an 18-item measure that assesses the manner in which clinicians deliver exposure therapy. Two subscales assess the frequency with which therapists use 10 intensive exposure techniques (e.g. encouraging exposure to the most feared situation) and eight therapist safety behaviors (e.g. imaging rather than actually facing feared situations). These two subscales are called the Intensive Exposure subscale and the Coping Exposure subscale, respectively. Items were rated on a 5-point Likert-type scale ranging from 0 (never use) to 4 (always use). Past research has found the internal consistency for the ETDS Intensive Exposure subscale (α = .86) and the ETDS Coping Exposure subscale (α = .88) to be good (Deacon et al., 2023). Only the primary item of interest (imagining rather than actual exposures) and three select items from the Intensive Exposure subscale for context were used in the current study (see Appendix).

#### Therapist Beliefs about Exposure Scale (TBES; Deacon et al., 2013).

The TBES is a 21-item questionnaire that assesses therapist beliefs about the safety, tolerability, and ethicality of exposure therapy. Participants use a 5-point scale ranging from 0 (disagree strongly) to 4 (agree strongly) to indicate their agreement with statements illustrating potential therapist concerns about exposure (e.g., “Most clients perceive exposure therapy to be unacceptably aversive”). Higher scores indicate greater negative beliefs about using exposure therapy to treat anxious clients. The TBES has previously demonstrated excellent internal consistency (α = .95), six-month test-retest reliability (r = .89), and good construct validity (Deacon et al., 2013). The internal consistency in the current sample was excellent (α = .91).

#### Beliefs Regarding Exposure Mechanism of Action Stewart et al., 2016).

To examine the theory underlying clinician use of exposure, clinicians were asked to rate their level of agreement ranging from 0 (disagree strongly) to 4 (agree strongly) with statements regarding exposure’s mechanism of action and the therapist’s role during exposure. To assess mechanism of action, therapists responded to four descriptions following the prompt “When clients improve as a result of exposure therapy it is because…” reflecting models of coping, habituation, acceptance, and inhibitory learning/cognitive change. In addition, clinicians were asked to use the same scale to indicate their level of agreement with four statements regarding whether the therapist’s role during exposures was to increase, decrease, maintain, or unrelated to the client’s anxiety. Endorsement of mechanism of action has been found to predict use of corresponding treatment techniques (Stewart et al., 2016).

### Planned Analyses

First, to examine the hypothesis that community therapists use of imaginal exposure reflects suboptimal use of exposure, a series of three repeated measures analyses of variance (RMANOVA) with planned contrasts were used to compare the frequency with which community therapists reported using a) imaginal exposures versus other therapeutic techniques to address anxious thoughts, b) direct imaginal exposure (i.e., repeating anxiety provoking thoughts) versus other responses to thoughts during exposure, and c) imaginal exposure as a replacement for in vivo exposure versus aspects of intensive exposure implementation. In addition, to examine the theory behind technique use, correlational analyses were conducted to examine the association of frequently endorsed techniques with beliefs about exposure and its implementation. Second, to examine the hypothesis that imaginal exposure is used to directly target worry thoughts in GAD, correlational analyses were conducted to examine the association between the frequency of technique use and the frequency with which the therapists reported treating youth with various anxiety disorders.

## Results

### Examining Imaginal exposure as sub-optimal exposure

#### Technique Use.

A RMANOVA indicated that the frequency with which therapists used the various techniques to address anxious thoughts (Table 1) differed signi cantly, *F* (6, 1008) = 20.27, *p* < .001, with planned contracts indicating that imaginal exposures were used more frequently than positive imagery and thought distraction, but less frequently than cognitive restructuring and replacing negative thoughts with positive thoughts (all p’s < .05). More than 50% of the therapists endorsed using every technique often or always, with the exception of positive imagery.

#### Technique Use During Exposure.

A second RMANOVA indicated that the manner in which therapists reported responding to anxious thoughts during exposure differed signi cantly (Table 1), *F* (4, 580) = 33.12, *p* < .001. Planned contracts indicated that repeating anxious thoughts (i.e., direct imaginal exposure) was endorsed less frequently than cognitive restructuring and separating from symptoms (all p’s < .05) but did not differ significantly from thought stopping or distraction. Only 27.0% of the therapists endorsed instructing patients to repeat anxiety provoking thoughts “often” or “always”, while 64.7% endorsed using cognitive restructuring “often” or “always”.

#### Exposure Implementation.

A third RMANOVA indicated that the frequency with which therapists implemented various aspects of exposure (Table 2) differed significantly, *F* (3, 432) = 85.71, *p* < .001, with planned contrasts indicating that therapists endorsed using imaginal exposures as a replacement for in vivo more frequently than they endorsed leaving the office to conduct exposure and helping patients face their greatest fear, but less often than completing in session exposures (all *p’s* < .05). More than 50% of the therapists endorsed using imaginal exposures rather than in vivo exposure “often” or “always”, while only 15.1% of therapists reported leaving the office to conduct exposure “often” or “always”.

#### Correlations between Beliefs and Technique Use.

Correlations between exposure implementation items and therapist beliefs are presented in Table 2. More frequent use of cognitive restructuring, thought stopping, and distraction was associated with more negative beliefs about exposure (higher score on TBES), belief that youth learned to cope with anxiety through exposure, as well as beliefs that the therapist’s role is to decrease (or not unrelated to) anxiety during exposure. Use of thought stopping and distraction were also negatively correlated with the belief that youth benefited from exposure through habituation. In contrast, use of repeating upsetting thoughts was associated with more positive beliefs about exposure (lower score on TBES), endorsement of a habituation view of exposures, and the view that the therapists’ role is to increase (and not decrease) anxiety during exposures.

#### Relation to GAD.

Correlations between the frequency with which clinicians treated various anxiety disorders and technique endorsement is presented in Table 3. The results indicate that use of imaginal exposure was significantly associated with the frequency of treating social phobia, GAD, panic, and separation anxiety disorder, but not agoraphobia, specific phobia, or OCD. Repeating negative thoughts was associated with the frequency of treating all anxiety disorders, with the exception of GAD. The frequency with which therapists reported treating GAD was most strongly associated with cognitive restructuring during exposure, followed by replacing negative thoughts with positive thoughts.

## Discussion

The data collected here provide some insight into the manner in which community therapists utilize imaginal exposures. Consistent with previous examinations of overall exposure use (Whiteside, Deacon, et al., 2016), imaginal exposure appears to be used infrequently and in a suboptimal manner, rather than as a targeted invention for patients with GAD. Although use of direct imaginal exposure is associated with belief in a habituation model of exposures, most of the strategies community therapists report using reflect a coping model of change. The findings have implications for assessment of treatment fidelity and for therapist training.

The current results suggest that although community therapists are more likely to use imaginal exposure as compared to in vivo or interoceptive exposure, imaginal exposure is likely implemented with low fidelity. To begin with, when imaginal exposure is more specifically defined as repeating anxiety provoking thoughts the endorsement drops by more than half, with more therapists reporting never using this technique than reporting using it often or always combined. Moreover, rather than implementing imaginal exposure to address anxiety provoking thoughts, therapists describe using imaginal exposure in lieu of in vivo exposure, perhaps because therapists are reluctant to have patients confront their greatest fear directly or because they find it difficult to implement in vivo exposures. Regardless, this manner of implementing imaginal instead of in vivo exposure is generally not recommended by experts and suggests that like rates of exposure in general, community therapist rarely use targeted imaginal exposure.

As with our previous investigations of exposure use, it is important to examine the implementation of imaginal exposure in the broader context of the other techniques community therapists are employing. Both as a general therapeutic technique and specifically during exposures, clinicians most frequently reported using cognitive restructuring, more often than imaginal exposure. Theoretically, cognitive restructuring is designed to reduce anxiety by helping patients see situations more accurately through challenging negative thoughts, rather than through confronting anxiety provoking stimuli. While these strategies may be less effective than exposure, they are prominently included in evidenced based treatment protocol and therapists cannot be faulted for using them (Whiteside, Sim, et al., 2020). However, therapists also reported using techniques that are not part of CBT manuals, such as replacing negative thoughts with positive thoughts, thought stopping, and distraction during exposure, at greater or similar frequency as IE. These strategies likely serve to encourage patients to avoid thoughts rather than challenge them through cognitive or exposure strategies. As such, although therapists do report using directed imaginal exposures, this technique likely accounts for a small percentage of the overall session time and therapeutic content.

Further understanding of the quality of imaginal exposure comes from examination of the therapists’ beliefs about therapeutic mechanisms of action. As would be expected, use of actual imaginal exposure (repeating anxiety provoking thoughts) was associated with a view that exposures work through habituation to anxiety provoking stimuli and the therapist should not try to reduce the patient’s anxiety. However, therapists were much more likely to report using cognitive restructuring and equally as likely to report using thought stopping and distraction during exposure than repeating anxious thoughts. Each of these techniques were related to a view of exposure as teaching youth that they can cope with anxiety and that the therapist’s role during an exposure is to help decrease anxiety. In fact, thought stopping and distraction during exposure were negatively related to belief in the habituation model. As a result, the majority of technique use by therapists during exposure is antithetical to use of repeating thoughts with the goal of habituation or inhibitory learning. Moreover, the use of each of the non-exposure strategies was related to negative views of exposure in general. In summary, even if use of direct imaginal exposure is associated with habituation, the majority of what therapists report implementing is at odds with this goal, adding support to the hypothesis that imaginal exposure is used instead of more challenging forms of exposure.

Finally, the current study found no support for the theory that community therapists are choosing to implement imaginal exposure to directly target worry thoughts in GAD. Although, not examined statistically, the use of imaginal exposure was similarly related to treating GAD, social phobia, panic and separation anxiety disorder. In fact, GAD was the only anxiety disorder not significantly associated with the more clearly defined direct use of imaginal exposure consisting of repeating anxiety-provoking thoughts. Rather, the treatment of GAD seems to be more associated with efforts to correct (cognitive restructuring) or suppress (replacing negative with positive thoughts) worry thoughts. And as we have seen these techniques are associated with helping children cope with anxiety, rather than an exposure-based habituation or learning model.

The current study has a number of limitations. First and foremost, the present analyses are a post hoc examination of existing data rather than a priori predictions. As such, the data collection was not designed to directly answer the questions posed and the conclusions are based on associations rather than direct testing. Regarding the data collection, as with the original study from this data set, technique use was based on endorsement of brief statements, and therapists may have interpreted those differently which would affect the accuracy with which they described their practice. This discrepancy is likely on display with the differences in endorsement of imaginal exposure versus repeating anxiety-provoking thoughts. In addition, the current sample was limited to clinicians in a single state and needs to be replicated in other regions.

Despite these limitations, the current study provides insight into the use of imaginal exposure by community therapists. As would be expected based on known patterns regarding the use of exposure in general (Whiteside, Deacon, et al., 2016), the current study suggests that community therapists generally use imaginal exposure in lieu of more intensive forms of exposure and as part of a treatment package generally aimed at helping youth with anxiety disorders cope, rather than to disconfirm anxiety provoking beliefs through exposure. As such, while use of imaginal exposure may be a step toward accepting and implementing exposure (Whiteside et al., 2022), it is by itself inadequate and unlikely to significantly improve outcome. As such, these findings suggest that dissemination efforts that increased use of imaginal exposure, likely failed to improved outcome due to insufficient training and fidelity, rather than shortcomings of the treatment itself. In terms of training, instruction in exposure must highlight the importance of in vivo exposure and it is possible that instruction in imaginal exposure may, without proper support, impede the use of in vivo exposure if therapist think that imaginal exposure is an acceptable substitution. In general, these findings speak to the importance focusing training on the intricacies of implementing exposure well.

## Figures and Tables

**Table 1:** Therapist Endorsement of Techniques to Treat Childhood Anxiety Disorders

	*M (SD)*	Never	Rarely	Moderately	Often	Always
**Technique Use**						
Cognitive Restructuring	3.97 (0.93)*	2.3%	2.3%	22.7%	40.9%	31.8%
Replace Negative Thoughts with Positive Thoughts	3.96 (1.13)*	4.0%	7.9%	16.4%	29.9%	41.8%
Mindfulness Techniques	3.79 (1.00)	2.8%	5.6%	27.1%	37.3%	27.1%
*Therapist Imaginal Exposure*	*3.76 (0.64)*	*0.0%*	*0.0%*	*36.0%*	*52.2%*	*11.8%*
Thought Stopping	3.64 (1.13)	7.3%	7.3%	18.6%	44.6%	22.0%
Thought Distraction Techniques	3.50 (1.20)*	10.3%	8.0%	24.6%	35.4%	21.7%
Positive Imagery	3.14 (1.25)*	14.1%	13.6%	28.2%	29.4%	14.7%
**Technique use During Exposure**						
Cognitive Restructuring	3.73 (1.16)**	6.0%	8.0%	21.3%	34.7%	30.0%
Separate Symptoms	3.10 (1.27)**	15.3%	18.0%	24.0%	29.3%	13.3%
Thought Stopping	2.84 (1.35)	24.7%	16.0%	19.3%	30.0%	10.0%
*Repeat Upsetting Thoughts*	*2.56 (1.33)*	*30.4%*	*20.3%*	*22.3%*	*18.2%*	*8.8%*
Distraction	2.32 (1.11)	27.7%	33.8%	20.9%	14.9%	2.7%
**Exposure Implementation**						
In session Exposure	3.86 (0.87)***	0.0%	5.9%	30.1%	39.2%	24.8%
*IE rather than in vivo*	*3.54 (0.82)*	*0.7%*	*9.9%*	*36.8%*	*43.4%*	*9.2%*
Most Feared Item	3.21 (1.09)***	5.4%	19.6%	37.2%	24.3%	13.5%
Leave the Office	2.09 (1.15)***	38.2%	34.9%	11.8%	11.2%	3.9%

Note.

* Mean differs from Therapist Imaginal Exposure mean *p* < .05.

** Mean differs from “Repeat Upsetting Thoughts” mean, *p* < .05.

*** Mean differs from “IE rather than in vivo” mean.

**Table 2:** Correlation between Technique use During Exposure and Therapist Beliefs

	Cognitive Restructuring	Separate from symptoms	Thought Stopping	Repeat upsetting thoughts	Distract
TBES	.30*	.00	.34*	−.34*	.29*
Exposure Mechanism					
Cope	.50*	.15	.28*	−.14	.19*
Habituate	−.16	−.01	−.25*	.35*	−.26*
Accept	−.06	.10	.09	−.16	.03
Disconfirm	.11	−.01	−.02	.08	.02
Therapist Role					
Increase	.08	.07	.01	.18*	.08
Decrease	.33*	.01	.28*	−.33*	.32*
Unrelated	−.21*	−.06	−.18*	.14	−.22*

* p < .05. TBES = Therapist Beliefs about Exposure Scale.

**Table 3:** Correlations between Technique use and frequency of diagnoses treated.

	Frequency treating						
	Soc	GAD	Pan	Ag	Pho	Sep	OCD
**Technique Use**							
Cognitive Restructuring	.18*	.28*	.23*	.07	.07	.04	.11
Replace Negative Thoughts with Positive Thoughts	.15	.30*	.17*	−.03	−.01	.13	.04
Mindfulness Techniques	.19*	.25*	.06	.04	−.02	.20*	.25*
*Therapist Imaginal Exposure*	.22*	.17*	.20*	.11	.11	.16*	.10
Thought Stopping	−.00	.23*	.10	−.09	−.05	.06	−.02
Thought Distraction Techniques	.12	.21*	.17*	−.12	−.04	.02	.01
Positive Imagery	.16*	.20*	.18*	.04	.16*	.12	.11
**Technique use During Exposure**							
Cognitive Restructuring	.04	.32*	.08	−.05	.00	.10	.11
Separate Symptoms	.18*	.15	.11	−.01	.09	.19*	.26*
Thought Stopping	.03	.19*	.06	−.12	−.05	.05	−.01
*Repeat Upsetting Thoughts*	.21*	.02	.22*	.25*	.21*	.19*	.19*
Distraction	−.01	.02	.00	−.12	−.11	−.06	−.01

*. Correlation is signi cant at the 0.05 level (2-tailed).

## Data Availability

Data is available upon request.
